# Hepatitis B Virus Infection among Japanese Immigrants and Descendants: The Need to Strengthen Preventive and Control Measures

**DOI:** 10.3390/v14051085

**Published:** 2022-05-18

**Authors:** Luiz Henrique Ferraz Demarchi, Larissa Melo Bandeira, Deborah Ledesma Taira, Marina Castilhos Souza Umaki Zardin, Mary Luizia Ibanhes, Ana Olivia Pascoto Esposito, Larissa Domingues Castilho de Arruda, Crhistinne Cavalheiro Maymone Gonçalves, Sabrina Moreira dos Santos Weis-Torres, Gabriela Alves Cesar, Rivaldo Venâncio da Cunha, Tayana Serpa Ortiz Tanaka, Marco Antonio Moreira Puga, Grazielli Rocha de Rezende, Roberta Barbosa Lopes, Silvia Naomi de Oliveira Uehara, João Renato Rebello Pinho, Flair Jose Carrilho, Michele Soares Gomes-Gouvêa, Ana Rita Coimbra Motta-Castro

**Affiliations:** 1 Laboratório Central de Saúde Pública de Mato Grosso do Sul/SES/MS, Campo Grande 79080-320, Brazil; deborah.taira@saude.ms.gov.br (D.L.T.); ninaumaki@gmail.com (M.C.S.U.Z.); mlibanhes@yahoo.com.br (M.L.I.); ana.esposito@saude.ms.gov.br (A.O.P.E.); 2 Laboratory of Clinical Immunology, Federal University of Mato Grosso do Sul, Campo Grande 79070-900, Brazil; weistorres.sms@gmail.com (S.M.d.S.W.-T.); g.alves.cesar@gmail.com (G.A.C.); tayeto@hotmail.com (T.S.O.T.); marco.m.puga@gmail.com (M.A.M.P.); grazielli_r@hotmail.com (G.R.d.R.); si.uehara@hotmail.com (S.N.d.O.U.); 3 Secretaria de Estado de Saúde de Mato Grosso do Sul, Campo Grande 79031-350, Brazil; larissa.castilho@saude.ms.gov.br (L.D.C.d.A.); crhismay@gmail.com (C.C.M.G.); 4 Instituto Oswaldo Cruz, Fiocruz, Rio de Janeiro 21040-900, Brazil; rivaldo_venancio@uol.com.br; 5 Secretaria Nacional de Vigilância em Saúde SVS, Ministério da Saúde, Brasília, Distrito Federal 70740-610, Brazil; robertabl@yahoo.com; 6 Hospital das Clínicas, Faculdade de Medicina, Universidade de São Paulo, São Paulo 05403-000, Brazil; jrrpinho@usp.br (J.R.R.P.); fjcarril@usp.br (F.J.C.); gomesmic@yahoo.com.br (M.S.G.-G.); 7 Fiocruz Mato Grosso do Sul, Fundação Oswaldo Cruz, Campo Grande 79081-746, Brazil

**Keywords:** Hepatitis B, epidemiology, seroprevalence, genotype

## Abstract

This cross-sectional study aimed to investigate the prevalence and risk factors of Hepatitis B virus infection among Japanese immigrants and their descendants from São Paulo (SP), and to verify the occurrence of occult hepatitis B and coinfection with HCV, Delta, and HTLV. All samples (*n* = 2.127) were tested for HBV serological markers by electrochemiluminescence. HBsAg and/or total anti-HBc positive samples were tested for HBV DNA by real-time PCR, and genotyped by sequencing using the Sanger methodology. The prevalence rate of HBV exposure was 13.4% (CI 95%: 11.9–14.9%), and 22 (1.1%) were HBsAg positive. A high rate of susceptibility to HBV infection was found (67.4%; CI 95%: 65.4–69.4%). In contrast, only 19.2% (CI 95%: 17.6–20.9%) presented a serological profile analogous to that elicited by Hepatitis B vaccination. HBV isolates (*n* = 8) were classified as genotypes HBV/B1 (62.5%), HBV/C2 (12.5%), HBV/F1b (12.5%), and HBV/A1 (12.5%). Hepatitis B vaccination strategies and educational measures to control this infection should be considered.

## 1. Introduction

Hepatitis B virus (HBV) is an important public health problem. The World Health Organization (WHO) estimates that 1/3 of the global population has serological evidence of HBV infection. Of these, 296 million are chronic carriers, with 820.000 deaths from liver complications related to chronic infection per year [1]. Among individuals chronically infected with HBV, 75% live in Asia, and 25% die from liver complications resulting from infection [2]. 

Currently, Brazil and Japan are considered countries of low endemicity for HBV infection, with a prevalence of HBsAg less than 2% and 1%, respectively [3,4]. However, the control and elimination of this viral infection has been a challenging task for both countries, given certain regions and populations experience a high prevalence of HBV infection [5].

In Japan, decreases in HBsAg carrier rates nationally have been observed as a product of medical and public health precautions, such as improvement of sanitary conditions and administration of Hepatitis B immunoglobulin and/or Hepatitis B vaccination to neonates of HBsAg-positive mothers. By contrast, the rate of HBsAg carriers in the Okinawa prefecture was found to be substantially higher, such that the region was declared endemic for HBV in a survey conducted by Hayashi et al. from 1968 through the 1980s [6].

In Brazil, the vaccination against Hepatitis B has been applied since the end of the 90s; this measure has been part of the vaccination schedule for children and also some specific population groups [7]. Nowadays, this vaccine is available in all health facilities to the whole population regardless of age and/or vulnerability conditions [8]. The coverage of vaccination in Brazil varies in different population groups. In a systematic review of the epidemiology of HBV in the 21st century conducted by Souto et al., the prevalence of vaccine-induced protection profiles (anti-HBs positive only) varies from 18% in the state of Pará (North), to 92.7% in the state of Bahia (Northeast) [5].

The immigration contingent coming from Japan to Brazil from 1908, as a result of a bilateral contract between Brazil and Japan, today reflects approximately 1.5 million Japanese descendants living in Brazil, considered the country with the largest amount of Japanese people outside of Japan. The majority of this group resides in the state of São Paulo, which received the largest contingent of Japanese immigrants, followed by Paraná, Mato Grosso do Sul, and Pará [9]. After their arrival in Brazil, one of the most serious problems that the first Japanese immigrants faced was the difficulty of accessing medical and health care [10,11].

Considering the high number of Japanese descendants in Brazil, the prevalence of HBV and HDV infections found in Asia and in some regions of Japan, the population exchange between these two countries, and the scarcity of studies in this population, this study aimed to investigate seroepidemiological and molecular aspects of HBV infection; its associated factors; and HBV co-infection with HDV, HCV, and HTLV among Japanese immigrants and their descendants residing in the Metropolitan region of São Paulo, SP.

## 2. Materials and Methods

This cross-sectional study was conducted among Japanese immigrants and their descendants living in São Paulo, Brazil. Between July 2017 and December 2017, participants were recruited from the five main Okinawan associations in the state of São Paulo (named A to E) from a study investigating HTLV (Human T-cell Lymphotropic Virus) prevalence [12]. The study population included male and female subjects who were Japanese immigrants, descendants of Japanese immigrants, or have a family relationship with these populations. The individuals were informed in detail about the research objectives and the confidentiality of the data. 

Informed written consent was obtained, after a detailed explanation of the study, at the time of sampling from all participants or their legal guardians, in case of individuals under age 18. The study protocol was approved by the Ethics Committee of Federal University of Mato Grosso do Sul (3.415.536 CAAE 15432319.3.0000.0021), and all participants gave a written informed consent (IC).

The participants were interviewed to obtain information on sociodemographic characteristics, HBV vaccination, and work-related and other risk behaviors [10]. Of the original 2.139 participants from the previous HTLV study, 2.127 who authorized future research in IC forms were included in the present investigation. 

Serum samples from 2.127 participants were screened for serological markers of HBV (HBsAg, total anti-HBc anti-HBs), HCV (anti-HCV), and HTLV (anti-HTLV-1/2) infections by electrochemiluminescence assay (ECLIA) following the manufacturer’s guidelines (Elecsys-cobas-Roche^®^). HBsAg-positive samples were tested for HBeAg, anti-HBe, and anti-HBc IgM by electroimmunoassay (ECLIA) using the Cobas1 e 601 Analyzer (Roche Diagnostics, Mannheim, Germany) following the manufacturer’s guidelines; anti-HCV-positive samples were confirmed by “line immunoassay” (INNO-LIA III HCV Ab, Innogenetics, Belgic). 

Current HBV infection was defined as a positive HBsAg test result. HBV exposure was defined as a positive total anti-HBc and/or HBsAg test result. The HBV-vaccination-like profile included all participants with the anti-HBs positive result (≥10 mIU/mL) combined with negative total anti-HBc and HBsAg results. Participants lacking total anti-HBc, anti-HBs, and HBsAg were considered serologically susceptible to HBV infection. Twelve samples from twenty-two positive HBsAg individuals were tested for the qualitative detection of total antibodies to hepatitis D virus, using a commercial chemiluminescence immunoassay (CLIA), according to the manufacturer’s instructions (LIAISON^®^ XL, Murex, DiaSorin, Saluggia, Italy). 

All HBsAg and/or total anti-HBc positive samples were retested, and subjected to HBV DNA detection and quantification by real-time PCR (qPCR). HBV DNA was extracted from serum samples using an Abbott mSample Preparation System DNA kit in an automated platform. After extraction, we proceeded with a real-time PCR in the Abbott m2000rt^®^ System, targeting the S-gene (surface antigen), given it is a highly conserved and specific HBV region that allows the detection of genotypes A to H. The limit of detection of the Abbott RealTime HBV is 10 IU/mL, the upper limit of quantification of the Abbott RealTime HBV is 1 billion IU/mL, and the lower limit corresponds to the detection limit.

Samples with detectable HBV DNA were submitted to HBV genotype/subgenotype identification. To achieve this, a fragment (1270 bp) of the overlapped S/Pol genes was amplified and sequenced as previously described [13]. HBV sequences characterized in this study were aligned by the ClustalW program implemented in BioEdit software [14], with 306 sequences available in the GenBank database of different HBV genotypes/subgenotypes originating from several countries. The phylogenetic tree was inferred using the Bayesian Markov Chain Monte Carlo (MCMC) method implemented in the BEAST package v.1.8.3 under a relaxed molecular clock, and GTR + G + I as the nucleotide substitution model; MCMC was run for 20 million generations, and trees were sampled every 2000 generations. The maximum clade credibility tree was summarized using TreeAnnotator v.1.8.3 [15], and the tree was visualized in FigTree v1.4.2. software (available at: http://tree.bio.ed.ac.uk/software/figtree, accessed on: 5 August 2021).

To compose the database, the questionnaires from 2127 participants included were analyzed to obtain sociodemographic, clinical, and behavioral information, and factors associated with HBV transmission, including: age; sex; history of residence in Japan; blood transfusion; surgery; unprotected sex; and whether the patient had ever been diagnosed with any sexually transmitted infection.

Data analysis was performed in the statistical software Stata SE, version 13 (StataCorp LP, College Station, TX, USA). This study used the chi-squared test (χ^2^) or the Fisher’s exact test (for categorical variables) to assess differences between proportions, and determine *p* values (two-tailed). The prevalence rate of HBV exposure (any HBV marker: HBsAg positive; HBsAg/total anti-HBc positive; anti-HBc alone positive; anti-HBc/anti-HBs positive) and a 95% confidence interval (CI) were calculated. Odds ratios and 95% confidence intervals (CI) were used to verify potential predictors of HBV infection and/or exposure (presence of anti-HBc and/or HBsAg markers). Variables with a *p*-value of less of 0.20 were included in multiple linear regression analysis. The selection of variables for the final model was performed stepwise, according to the number of events per variable (EPV). A Hosmer–Lemeshow test was used to assess goodness-of-fit, choosing the best regression equation [16]. A *p*-value less than 0.05 was considered statistically significant. Individuals with anti-HBs alone (≥10 mIU/mL) were excluded from the HBV associated factors analysis, since it probably indicates an HBV-vaccination-like profile (*n* = 409). 

## 3. Results

A total of 2.127 individuals, Japanese immigrants, descendants of immigrants, and individuals with a family relationship with these populations residing in the metropolitan region of São Paulo were enrolled in this study. Most of this population (59.8% female and 40.2% male) was over 60 years old (41.9%), and reported more than twelve years of formal education (44.9%). The majority of participants were either married or divorced/widowed (74.5%), and reported receiving more than US$500 (65.1%) as monthly income. Among this cohort, 47.3% were sons or daughters of Japanese people, of which 87.5% were of Okinawan descendant with no history of living in Japan (61.4%). In addition, 93.2% reported no blood transfusion; 65.5% had never had surgery; 90.1% had never been diagnosed with a sexually transmitted infection; 96.9% had never had any accidental contact with the blood of others; 59.4% reported they had never shared sharp objects; 83.7% reported no regular use of condoms; and 95.2% were born in São Paulo. Socio-demographic characteristics, sexual behavior, using or sharing objects, and origin are shown in Table 1. 

A low frequency was found for several variables among the studied population, including: tattooing (7.2%); piercing (4.0%); having ever had a homosexual relationship (0.7%); previous incarceration (0.33%); injecting drug usage (0.1%); and non-injectable drug use (2.2%). 

Of 2.127 Japanese immigrants, their descendants, and non-Japanese family members, the prevalence rate of HBV exposure was 13.4% (284; 95% CI: 11.97–14.86). Among them, six (0.3%) were positive for HBsAg only, and sixteen (0.8%) were HBsAg- and total anti-HBc-positive, resulting in an HBsAg-positive “overt” HBV infection rate of 1.1%. All of them were anti-HBc IgM-negative. 

Two hundred and five (9.6%) had been infected with HBV, and had developed natural immunity (total anti-HBc-positive and anti-HBs-positive), and 2.7% (57/2.127) were positive only for total anti-HBc. Most of them (67.4%) were susceptible to HBV infection (had no HBV serological marker), and only 19.2% (409/2.127; 95% CI: 17.6–20.9) had serological evidence of previous Hepatitis B vaccination (isolated anti-HBs ≥ 10 mIU/mL) (Table 2). Among them (409/2.127), the majority were women (265/409); the highest prevalence rate of an HBV-vaccination-like profile occurred in the age group of less than 45 years (63.1% 258/409); and with increasing age, there was a decrease of vaccinated individuals.

**Figure 1:**
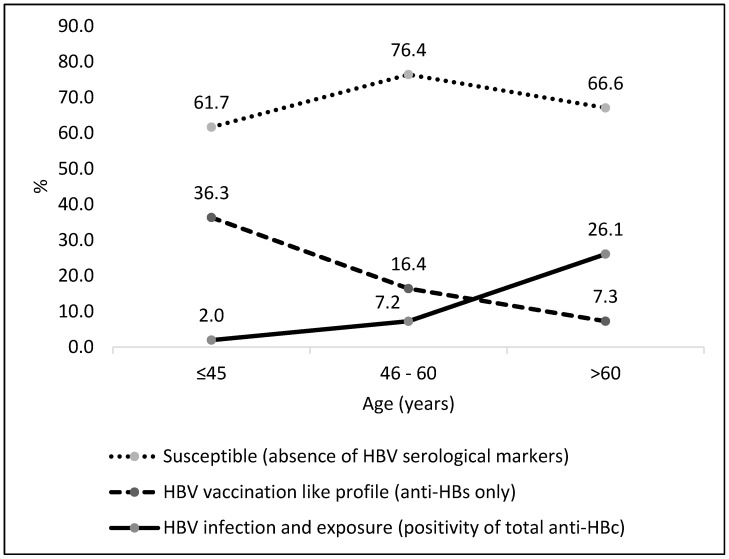
HBV serological profile among Japanese immigrants and their descendants according to age (years), Brazil (*n* = 2.127).

As shown in Figure 1, the group aged ≥60 years had the highest prevalence of HBV exposure (26.1%), and the group aged ≤45 years presented the highest prevalence of anti-HBs alone (36.3%). On the other hand, the number of susceptible individuals was high in all age groups.

Considering individuals with a vaccination serological profile (409/2.127), those whose grandparents were Japanese immigrants represent the generation with the highest rate of HBV vaccination (43.8%; 179/409), followed by those who were a son/daughter (36.9%; 151/409) of immigrants, and individuals who were a great-grandson/granddaughter (12.2%, 50/409) of immigrants. The lowest HBV vaccination rate was observed among first-generation Japanese immigrants (7.1%; 29/409). 

In a univariate analysis, seroprevalence of HBV was associated with: individuals who were >46 years old; originally from Association C, D, and E; married or single; Japanese immigrants or sons of Japanese immigrants; of Okinawan descendant; had lived in Japan; had less than 9 years of education; received one to three minimum wages; had at least one ^b^(OR = 3.46; *p* = 0.001); being originally from Association C (OR = 2.63; *p* = 0.000), Association D (OR = 2.96; *p* = 0.000), or Association E (OR = 2.74; *p* = 0.000); being a son of Japanese immigrants (OR = 2.39; *p* = 0.002); and being a first-generation Japanese immigrant (OR = 6.79; *p* = 0.000) were independently associated with positivity for Hepatitis B exposure (Table 3).

Among all HBsAg-positive samples, anti-HTLV-1 was detected in three (13.0%) of the subjects, and no samples tested positive for anti-HCV. Out of the 16 HBsAg-positive and 4 total anti-HBc-positive tested samples, no one was positive for total anti-HD antibodies (Table 4).

HBV DNA was detected in 10/22 (45.4%) HBsAg-positive samples, and in 13/263 (4.9%) total anti-HBc positive samples without HBsAg characterizing HBsAg-negative “occult” Hepatitis B infection (OBI). Demographic, serological and virological characteristics of these cases are detailed in Table 5. Of the 13 occult HBV infections, 5 (36.5%) were positive for anti-HBc only, and 8 (61.5%) were positive for total anti-HBc and anti-HBs. Considering all participants with HBV DNA detectable, the prevalence of HBV active/persistence infection was 1.6%. No one was positive for anti-HCV, and only one (OKW487) was positive for anti-HTLV-1/2.

**Figure 2:**
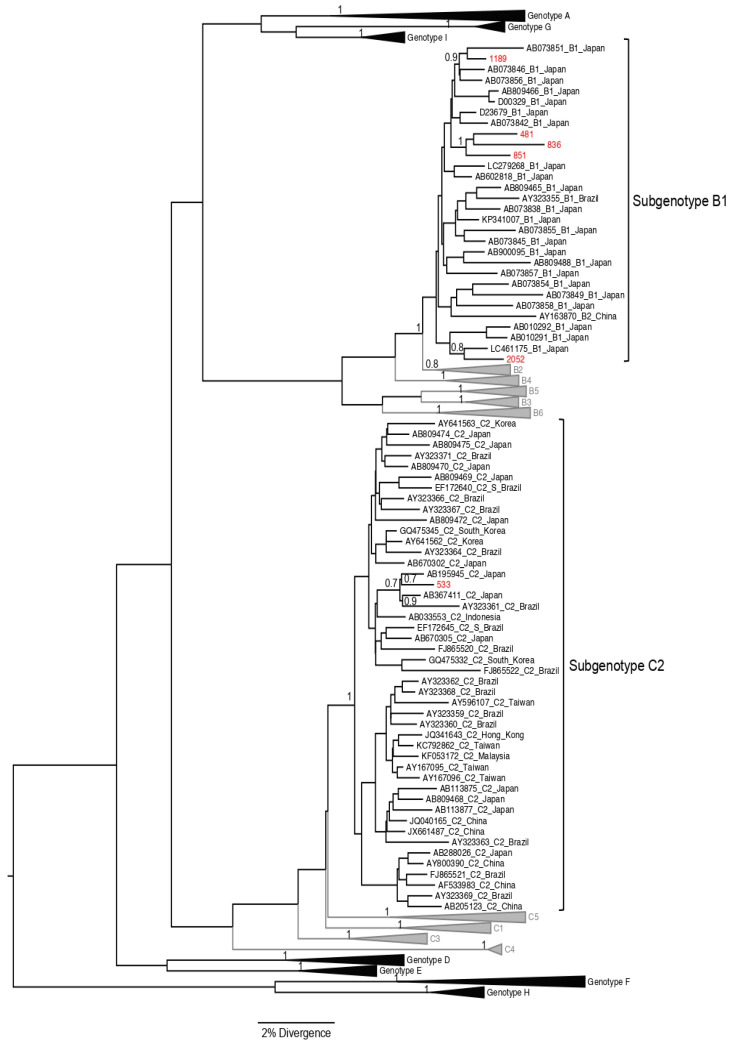
The maximum clade credibility (MCC) tree estimated by Bayesian analysis of S/POL sequences with 1270 bp of HBV strains characterized from Japanese immigrants and descendants living in São Paulo, Brazil. For better visualization, only clades of HBV/B1 and C2 are shown in this figure. Sequences characterized in this study are in red; sequences obtained from GenBank (*n* = 306) are indicated by their corresponding accession number, genotype, and geographic origin. The values of posterior probability are shown for key nodes.

**Figure 3:**
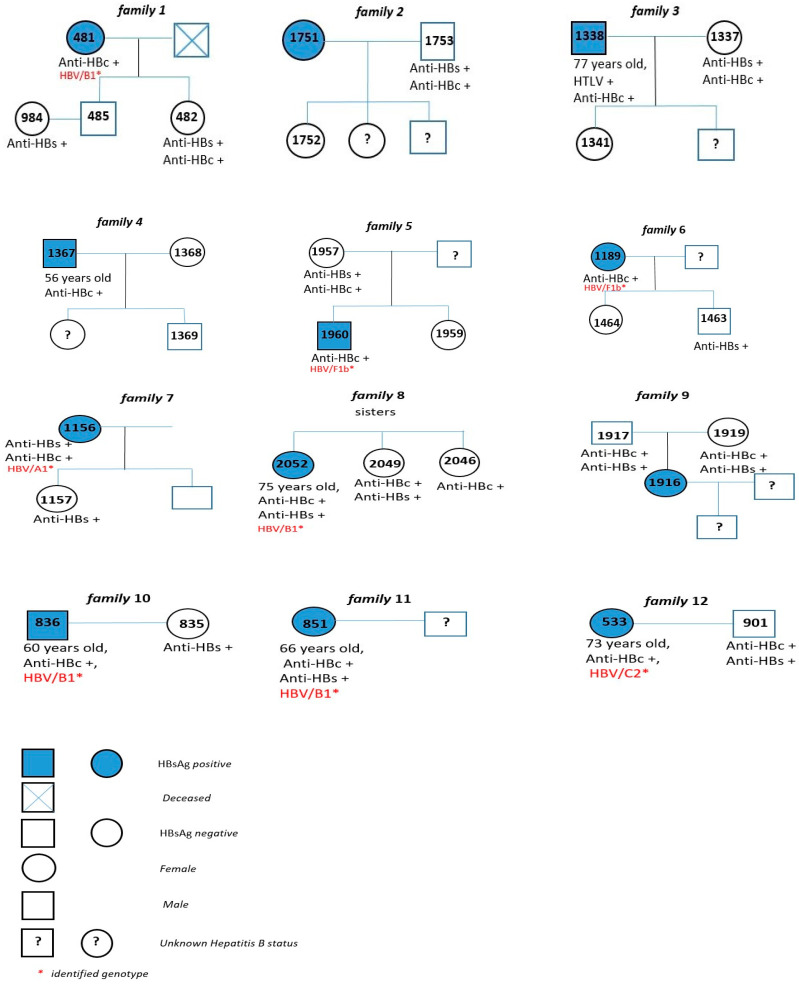
Pedigree of 12 families of index cases (HBsAg-positive) under study, São Paulo—SP.

**Figure 4:**
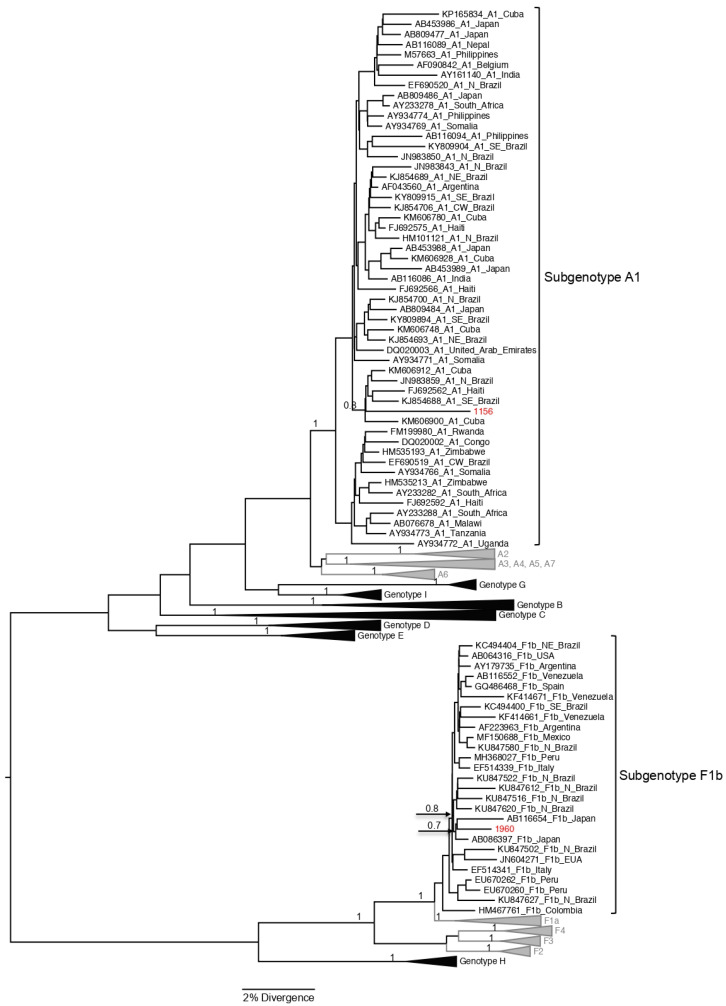
The maximum clade credibility (MCC) tree estimated by Bayesian analysis of S/POL sequences with 1270 bp of HBV strains characterized from Japanese immigrants and descendants living in São Paulo, Brazil. For better visualization, only clades of HBV/A1 and F1b are shown in this figure. Sequences characterized in this study are in red; sequences obtained from GenBank (*n* = 306) are indicated by their corresponding accession number, genotype, and geographic origin. The values of posterior probability are shown for key nodes.

The S/Pol region was amplified and sequenced from 8 of the 26 samples with HBV DNA detectable, and all of them were from cases with positivity to HBsAg. Subgenotype B1 was the most frequent (62.5%; 5/8) among the study population; four of these cases were Japanese immigrants, and one was a Japanese son. Three HBV/B1 sequences were closely related, but there was not any familiar relationship among these HBV carriers (Figure 2 and Figure 3). As reported by Carrilho et al., families represent an excellent model to study transmission mechanisms through people exposed to environmental characteristics [17]. Subgenotypes A1, C2, and F1b (one case each) were also found (Figure 2 and Figure 3). HBV/C2 was isolated from a Japanese immigrant and in a phylogenetic tree grouped together with HBV/C2 sequences isolated in Japan. HBV/A1 was also isolated from a Japanese son, and grouped closely with HBV/A1 sequences isolated in Brazil previously (Figure 4).

Subgenotype F1b (Figure 4) also was isolated from a Japanese son and the sequence grouped with two sequences isolated from Japanese patients in Okinawa (GenBank AB086397 and AB116654) [18]. The cluster of these three HBV/F1b sequences was closely related with a cluster with four F1b sequences isolated in North/Northwest regions of Brazil. 

## 4. Discussion

To our knowledge, this is the largest HBV epidemiological study involving this population ever performed in Brazil considering the number of Japanese immigrants. The prevalence of HBV serological markers describes the endemicity of Hepatitis B in this population group, and may provide necessary information to guide prevention and control policies improving public health.

In this study, a high prevalence rate of HBV exposure (13.5%: 95% CI: 12.1–14.9%) was found, which was high when compared to the prevalence found in blood prime donors (3.0%) from the same region [19]; this information reinforces the idea that Brazil has a heterogeneous distribution of HBV exposure despite the country’s low endemicity [5,20]. This rate is as high as another survey performed in Okinawa in the 1980s in which the prevalence of exposure varied from 3.0% in people aged 0–9 years old to 91.2% in people aged 30–39 years old [21]. This supports the hypothesis that immigrants of first generation were infected in their childhood in Japan. 

HBV can persist in an “overt” or in a long term “occult” state, depending on the detection of viral markers. The overt state, known as HBV chronic infection, is defined as persistence of HBsAg for six months or more after acute infection with HBV. The occult HBV infection is defined as the presence of replication-competent HBV DNA in the liver, in the absence of detectable serum HBsAg. Though the prevalence of HBV exposure was high, the prevalence of chronic HBV infection (1.1%), even when we consider the prevalence of HBV persistence (1.8%), was considered low, similar to other countries with low endemicity [5,22]. As reported by Rossi et al., in a study involving immigrants and refugees, chronic HBV seroprevalence was found to be high for migrants from East Asia (11.3% (95% CI: 10.3–12.4%)) when compared to migrants from Central Asia and South Asia (5.8% (95% CI: 4.3–7.9%)), and migrants from Latin America and the Caribbean (1.7% (95% CI 1.1–2.7%)) [23]. The CDC in the United States and the Canadian Collaboration for Immigrants and Refugee Health strongly recommends that all immigrants originating from countries with a seroprevalence of HBV higher than 2% should be screened for chronic HBV infection and prior immunity to HBV, and vaccinated if found to be susceptible [24,25]. The role of chronic HBV infection in the development of hepatocellular carcinoma (HCC), a global public health issue, is undisputed. Globally, chronic infection with HBV is the most common type of liver cancer, accounting for 85–90% of the cases. Globally, HBV chronic infection is the most prominent risk factor for HCC development, and it was responsible for 33% of liver cancer mortality, followed by alcohol (30%), HCV (21%), and other causes (16%), with substantial variation among countries in the underlying etiologies. HCC cases associated with chronic Hepatitis B infection account for 60% of cases in Asia and Africa, and 20% of cases in the West [26,27]. In Brazil, in a study conducted among 884 compensated cirrhotic patients under a surveillance program in the largest referral center for hepatology in Brazil, it was observed that 50.0% (8/16) of patients with HBV infection and HCC were Brazilian East-Asian descendants with a family history of HCC [28,29]. 

About 5% of people who have chronic infection with HBV are affected by hepatitis D virus (HDV), which cannot occur in the absence of HBV. Co-infection of HDV and HBV is considered the most severe form of chronic viral hepatitis due to rapid progression towards hepatocarcinoma [30]. Epidemiological studies have revealed that the incidence of HDV infection is low in Japan, varying from 0.6 to 3%, and it is considered lower when compared to the rates reported in Africa, North America, Central America, South America, and Europe [31,32]. For hepatitis D, 12 HBsAg-positive samples were analyzed, and none were positive for total anti-HD antibodies. This information reinforces that HDV infection is uncommon among HBsAg carriers in Japan [31,33] and in Brazil, the latter of which was found by Lago et al. (2018) to have a 3.2% national prevalence, and a 1.7% prevalence in the Southeast region [34]. 

HBV/HTLV-1 co-infection was found in 13% of HBsAg-positive samples. This result is in concordance with the high prevalence of HTLV-1 infection observed among this population in a study conducted by Bandeira et al. (2021) [12]. Interestingly, despite HBV and HCV sharing overlapping routes of transmission, no cases of HCV infection were found in the present study. 

The association between HBV exposure and older age (>60 years old) found in this study has been reported previously [20,35,36]. It is noteworthy that most of the Japanese immigrants infected were over 60 years old, given Japanese immigration to Brazil has largely ceased since the mid-20th century. This association suggests that, with increasing age, the risk of acquiring HBV by exposure increases mainly because of the elevated HBV prevalence among the inhabitants of Okinawa, when compared to Japan’s national average observed two decades before contracted migration to Brazil. 

In addition, this age group had not been vaccinated against Hepatitis B when Brazil started vaccination campaigns against HBV infection among the youngest citizens, and consequently, this decreased the number of new cases of HBV infection in this younger cohort.

A high susceptibility rate to HBV infection (67.3%) and a low positivity index for the serological marker of vaccine immunity (19.2%) were found in this group population, probably because it is predominantly older and has not received the HBV vaccine, highlighting that vaccination strategies and educational measures to control this infection should be considered in this population group. Similar to findings reported in other studies, increasing age was associated with HBV exposure, suggesting that people who were born before the initiation of vaccination programs were more likely exposed to the risk of infection [20,36]. 

The prevalence found for OBI was 6.1%; this is considered high in comparison to another study conducted among blood donors performed in São Paulo in which the prevalence was 0.6% [37]. It has been shown in many studies that rates for OBI vary in different populations according to the endemicity of HBV infection. In a survey conducted in a population from the Brazilian Amazon region, the rate of OBI among anti-HBc-positive patients was 14.36% [38]; in another study of patients on hemodialysis in the Northeast of Brazil, the prevalence found was 2.3% [39].

As shown in Table 3, being a Japanese immigrant (OR = 6.56; *p* = 0.000) or son/daughter (OR = 2.31; *p* = 0.000) were found to be independent factors associated with HBV exposure. As previously described, Brazil is home to the largest Japanese population outside of Japan, and about half of these immigrants came from southern Okinawa after World Wars I and II. This may be inferred because, as reported by Sakugawa et al. in 1991 [40], the prevalence of HBV exposure in Okinawa was the highest in Japan until the 1960s, decreasing from 12.3% in 1968 to 7.5% in 1979–1981 [6], and 4.7% in 1983, as reported by Kashiwagi et al. [41], and to 1.7% in 1985, as reported by Kashiwagi et al. (1988) [42], among children (1–5 years old). This high prevalence, mainly among the elderly (over 60 years old), likely reflects the geographical origin of infected individuals mainly between 1917 and 1940, when 164.000 Japanese people migrated to Brazil; 75% of these immigrants settled in São Paulo, where most of the coffee plantations were located. HBV exposure decreased among participants with higher educational levels (>9 years of education), which might be due to awareness of prevention; this finding is supported by similar observations reported by Rezende et al., 2020 [43].

After multivariate analysis, being originally from Association C (OR = 2.47; *p* = 0.001), D (OR = 2.94; *p* = 0.000), or E (OR = 2.67; *p* = 0.000) was associated with positivity for anti-HBc. This finding could be inferred given the majority of people from these associations were >60 years old when compared with Association A and B (*p* = 0.000).

The distribution of HBV genotype may demonstrate different patterns of migration to the Americas, and reflect human migration from different areas into the studied region [44]. In this study, subgenotypes A1, B1, C2, and F1b were found.

Genotype A is distributed globally, and is the main genotype found in sub-Saharan Africa, North America, India, Northern Europe, and Western Africa [45,46]. In Brazil, the most common genotype is A, followed by genotypes D and F [45]. Among the population of Japanese immigrants and their descendants living in São Paulo studied, HBV subgenotype A1, B1, C2, and F1b were found. 

HBV/A1 was isolated from a Japanese son, and the sequence was related with HBV/A1 sequences isolated previously in Brazil, where this subgenotype is widespread. Genotypes B (classified into B1, B2, B4–B6, and quasi-subgenotype B3) and C (divided into C1, quasi-subgenotype C2, C3–C16) are found predominantly in East and Southeast Asia, Indonesia, and Oceania [46,47]. Subgenotype B1 is predominantly found in Japan; and C2 is found in Japan, Taiwan, China, and Southeast Asia [48]. All samples classified as subgenotype HBV/B1 (*n* = 5) were isolated from Japanese immigrants, except one (OKW2052), who was the child of a Japanese immigrant. This finding is in agreement with studies conducted in Okinawa and Tohoku that found a high rate of HBV/B, and another survey performed among HBV chronic patients in Okinawa that found a positivity of 86.9% of HBV/B [49,50]. It is interesting that three of these HBV/B1 sequences grouped in a well-supported monophyletic clade, suggesting a common origin of the HBV strains (Figure 2). Notably, among the five isolates that were classified as HBV/B1, three were from the same Okinawa association, emphasizing the importance of screening families of chronic Hepatitis B carriers to increase the chances of effective treatment, and reduce the spread of this infectious disease [51]. 

The singular sample classified as HBV/C2 was from a 75-year-old male Japanese immigrant. This genotype has been previously found in Japanese and Chinese people from South and Southeast Brazil, as reported by Clemente et al. [52]. Most reports of HBV/B and HBV/C are linked to Asian communities due to behavioral factors and/or route of exposure, as reported by Lago et al. [34]. As described by Sunbul (2014), geographic distribution of HBV genotypes may be related to the route of exposure. For example, HBV/B and HBV/C are more common in high-endemic regions, where they are spread by perinatal or vertical exposure, both considered important transmission routes of HBV [46]. 

In a study conducted by Lago et al. [53], genotype HBV/F, subdivided into four subgenotypes (F1–F4), is more frequent in Amerindian populations of Central and South America. Subgenotype F1 includes two clusters, 1a (with strains isolated in Central America) and 1b (with strains isolated in Argentina) [48,54]. In this study, the HBV/F1b was identified in one sample from a 52-year-old female descendant of a Japanese immigrant. This sequence was closely related (*p* value = 0.7) with F1b sequences isolated from two patients in a hospital in Okinawa, Japan (AB086397 and AB116654), one of whom was a 43-year-old male chronic HBV carrier [18]. In Brazil, subgenotype F1b shows a low prevalence, and was described in few cases [55]; however, a recent study found this subgenotype in a large number of cases in patients from Rio Branco city, Acre state, and the Northern region of Brazil [56]. There is neither enough information about the ancestral origins of the patients from the Northern region of Brazil [56] infected by HBV/F1b, nor epidemiological information about the possible infection of Japanese patients with HBV/F1b described by Kato et al. [18], and we cannot make conclusions about the origin of this subgenotype among the Japanese population.

This study has some limitations. First, as a cross-sectional study, the exposure and outcome were simultaneously assessed. For this reason, it is impossible to draw any conclusions on causality. Although this study included the most populous Associations of Japanese immigrants and their descendants, this group might not represent the population as a whole, and might be biased by volunteers. Self-reporting and recall-bias are also limitations of the present study. The lack of HBV vaccination records likely overestimated the frequency of susceptibility, given some HBV vaccinated individuals lose detectable levels of anti-HBs over time.

Despite these limitations, this work highlights the importance of promoting further investigation surrounding HBV and other infectious diseases in immigrant populations. More important than preventive methods are to plan health interventions and policies to prioritize allocation of resources for the improvement of health surveillance. The high susceptibility rate to HBV infection and the low positivity index for the serological marker of vaccine immunity found in this study highlight the urgent need for Hepatitis B vaccination strategies, educational measures, and screening strategies to control this infection. 

## Figures and Tables

**Table 1:** Characteristics of Japanese immigrants and their descendants living in São Paulo (*n* = 2.127).

Characteristics	N ^a^	%
Age (years)		
≤45	710	33.4
46–60	524	24.6
≥60	893	41.9
Gender		
Male	856	40.2
Female	1.271	59.8
Formal education (years) ^a^		
Illiterate	18	0.85
1–9	559	26.4
10–12	588	27.8
>12	949	44.9
Monthly household income * ^a^		
<1	121	6.5
1–3	565	28.7
>3	1.281	65.1
Marital status		
Single	544	25.6
Married, divorced, or widowed	1.583	74.4
Recruitment site		
Association A	709	33.3
Association B	302	14.2
Association C	440	20.7
Association D	344	16.2
Association E	332	15.6
Family heritage		
Japanese immigrant	316	14.9
Japanese son/daughter	1.007	47.3
Grandson and great-grandson of Japanese immigrant	750	35.3
Non-Japanese descendant	54	2.5
Okinawan Descendant		
No	265	12.5
Yes	1.862	87.5
History of residence in Japan		
No	1.305	61.4
Yes	822	38.7
Blood transfusion		
No	1.982	93.2
Yes	145	6.8
Surgery		
No	734	34.5
Yes	1.393	65.5
History of Sexually transmitted infection ^a^		
No	1.917	90.1
Yes	144	7.0
Shared sharp objects		
No	1.263	59.4
Yes	864	40.6
Accidental contact with blood of others		
No	2.061	96.9
Yes	66	3.1
Regular use of condoms ^a^		
No	1.576	83.7
Yes	308	16.3
Born in São Paulo		
No	1.153	54.2
Yes	974	45.8

* National minimum wage: during the study period, one minimum wage was approximately R$937.00 BRL (U$285.00 USD). ^a^ The total represents the number of individuals who answered the question.

**Table 2:** Prevalence of Hepatitis B virus serological markers among 2.127 Japanese immigrants residing in the Metropolitan region of São Paulo–SP.

Markers	*n*	%	95% CI ^1^
***Infected***			
*HBsAg-positive*	*22*	*1.1*	0.68–1.56
HBsAg only	6	0.3	0.13–0.62
HBsAg/anti-HBc	16	0.8	0.46–1.22
*Anti-HBc positive*	*262*	*12.3*	11.10–13.93
Anti-HBc only	57	2.7	2.07–3.46
Total anti-HBc/anti-HBs	205	9.6	8.45–10.96
**Any HBV infection marker**	**284**	**13.4**	**11.97–14.86**
***Not susceptible, possibly vaccinated***			
Anti-HBs only	409	19.2	17.61–20.96
***Not exposed, susceptible***	1434	67.4	65.40–69.40

^1^ Confidence Interval.

**Table 3:** Factors associated with Hepatitis B virus infection among Japanese immigrants residing in the metropolitan region of São Paulo, Brazil (*n* = 1.718).

Factors/Variables	HBV Exposure ^a^ N (%)		OR (95% CI)	*p*-Value	Adjusted OR ^c^ (95%CI)	*p*-Value ^b^
Age						
≤45	14/452	3.1	1	-	1	-
46–60	37/438	8.4	2.88 (1.53–5.42)	0.001	1.26 (0.62–2.57)	0.526
>60	233/828	28.1	12.25 (7.04–21.30)	0.000	3.46 (1.70–7.04)	0.001
Gender						
Female	160/1006	15.9	1			
Male	124/712	17.4	1.11 (0.86–1.44)	0.406	-	-
Marital status						
Separated/divorced	61/246	24.8	1	1	1	-
Married	202/1122	18.0	0.67 (0.48–0.92)	0.015	1.01 (0.68–1.51)	0.929
Single	21/350	6.0	0.15 (0.11–0.32)	0.000	1.17 (0.56–2.44)	0.674
Recruitment site						
Association A	40/366	10.9	1	-	1	-
Association B	69/563	12.4	1.14 (0.75–1.72)	0.539	1.40 (0.88–2.25)	0.150
Association C	41/237	17.3	1.70 (1.06–2.73)	0.026	2.63 (1.53–4.51)	0.000
Association D	74/269	27.5	3.10 (2.02–4.72)	0.000	2.96 (1.82–4.81)	0.000
Association E	60/283	21.2	2.19 (1.42–3.39)	0.000	2.74 (1.66–4.51)	0.000
Family heritage ^b^						
Grandchild/great-grandchild of Japanese	21/537	3.91	1	1	1	-
Japanese son/daughter	135/856	15.7	4.60 (2.86–7.38)	0.000	2.39 (1.38–4.12)	0.002
Japanese immigrant	127/287	44.2	19.50 (11.89–31.98)	0.000	6.79 (3.45–13.38)	0.000
Okinawan descendant						
No	22/210	10.48	1	-		
Yes	262/1508	17.3	1.79 (1.13–2.85)	0.013	1.07 (0.62–1.85)	0.802
History of residence in Japan						
No	109/1017	10.7	1		1	
Yes	175/701	24.9	2.77 (2.13–3.60)	0.000	1.21 (0.81–1.80)	0.339
Education (years) ^b^						
>9	152/1172	12.9	1		1	
<9	130/534	24.3	2.16 (1.66–2.80)	0.000	1.06 (0.74–1.53)	0.742
Income ^b^						
>3 minimum wage (0)	138/998	13.8	1		1	
1 to 3 minimum wages (2)	104/483	21.5	1.71 (1.29–2.27)	0.000	1.08 (0.76–1.54)	0.660
<1 minimum wage (3)	22/109	20.2	1.57 (0.95–2.60)	0.075	0.91 (0.51–1.62)	0.760
Piercing						
No	281/1663	16.90	1			
Yes	3/55	5.45	0.28 (0.87–0.91)	0.025 ^d^	0.50 (0.62–4.12)	0.524
History of surgery						
No	78/551	14.16	1			
Yes	206/1167	17.65	1.29 (0.98–1.72)	0.069	0.85 (0.59–1.19)	0.347
Tattooing						
No	271/1609	16.84	1			
Yes	13/109	11.93	0.67 (0.37–1.21)	0.184	0.78 (0.39–1.56)	0.484
Shared personal sharp objects						
No	187/1037	18.03	1		1	
Yes	97/681	14.24	0.75 (0.57–0.98)	0.039	1.02 (0.73–1.41)	0.906
Non-injectable drug usage						
No	281/1679	16.7	1			
Yes	3/39	7.7	0.41 (0.13–1.35)	0.145	0.39 (0.11–1.40)	0.150
Sexually transmitted infection (STI) ^b^						
No	255/1546	16.49	1		1	
Yes	29/124	23.39	1.54 (0.99–2.39)	0.051	1.20 (0.71–2.04)	0.495
Condom use ^b^						
Always	29/217	13.36	1			
Sometimes/never	248/1332	18.62	0.67 (0.44–1.02)	0.062	1.26 (0.75–2.11)	0.382
Anti-HTLV positivity						
No	252/1626	15.50				
Yes	32/92	34.78	2.90 (1.80–4.56)	0.000	1.32 (0.78–2.24)	0.297

95% CI: 95% confidence interval; OR: odds ratio; ^a^ HBV exposure was defined as a positive total anti-HBc and/or anti-HBs test result; ^b^ the total represents the number of individuals who answered the question; ^c^ adjusted for age and gender; ^d^ Fisher’s test.

**Table 4:** Demographic, serological, and virological characteristics of HBsAg- and/or HBV-DNA-positive Japanese immigrants and their descendants in São Paulo, Brazil (*n* = 22).

ID	Age (Years)	Recruitment Site	Gender	Serological Markers			HBV Viral Load (log)	HBV Subgenotype
				HBeAg	Anti-HBe	Anti-HTLV		
49 ^a^	63	A	M	Negative	Positive	Negative	<1.00	NP
481 ^a^	82	B	M	Negative	Positive	Negative	2.79	B1
533 ^a^	73	B	M	Negative	Positive	Positive	2.07	C2
815 ^b^	44	B	M	Negative	Positive	Negative	1.4	NP
836 ^a^	60	B	F	Negative	Positive	Negative	2.25	B1
851 ^a^	66	B	M	Negative	Positive	Negative	1.98	B1
1156 ^b^	69	B	M	Negative	Positive	Negative	1.57	A1
1189 ^a^	58	C	M	Negative	Positive	Negative	3.32	B1
1338 ^b^	77	C	F	Negative	Positive	Positive	ND	NP
1367 ^a^	56	C	F	Negative	Positive	Negative	ND	NP
1576 ^b^	71	D	M	Negative	Negative	Negative	ND	NP
1592 ^a^	73	D	F	Negative	Negative	Negative	ND	NP
1596 ^b^	68	D	M	Negative	Negative	Negative	ND	NP
1621 ^a^	67	D	M	NP	Positive	Positive	NP	NP
1646 ^c^	47	D	F	Negative	Negative	Negative	NP	NP
1677 ^a^	67	D	F	Negative	Positive	Negative	ND	NP
1751 ^b^	64	D	M	Negative	Negative	Negative	ND	NP
1916 ^c^	39	E	M	Negative	Negative	Negative	ND	NP
1922 ^a^	64	E	F	Negative	Positive	Negative	2.53	NP
1960 ^b^	52	E	F	Negative	Positive	Negative	2.69	F1b
1974 ^a^	66	E	F	Negative	Positive	Negative	1.6	NP
2052 ^b^	75	E	M	Negative	Positive	Negative	2.12	B1

^a^ Japanese immigrant; ^b^ Japanese son/daughter; ^c^ Japanese grandchild/great-grandchild; M, male; F, female; HBeAg, Hepatitis B e antigen; anti-HBe, antibodies against HBeAg; ND, not detected; NP, not performed (insufficient specimen volume for testing).

**Table 5:** Demographic, serological, and virological characteristics of HBV-DNA-positive/HBsAg-negative of Japanese immigrants and their descendants in São Paulo, Brazil (*n* = 13).

ID	Age (Years)	Recruitment Site	Gender	Serological Markers		HBV Viral Load (log)	Subgenotype
				HBeAg	Anti-HBe		
49 ^a^	63	A	M	Negative	Positive	<1.00	NP
168	42	A	M	NP	NP	<1.00	NP
317	70	A	F	NP	NP	1.66	NP
487	87	B	F	NP	NP	<1.00	NP
815 ^b^	44	B	M	Negative	Positive	1.4	NP
935	72	B	M	NP	NP	1.02	NP
976	66	B	M	NP	NP	<1.00	NP
1313	34	C	F	NP	NP	<1.00	NP
1604	64	D	F	NP	NP	1.67	NP
1922 ^a^	64	E	F	Negative	Positive	2.53	NP
1974 ^a^	66	E	F	Negative	Positive	1.6	NP
2087	75	E	F	NP	NP	<1.00	NP
2103	65	E	M	NP	NP	<1.00	NP

^a^ Japanese immigrant; ^b^ Japanese son/daughter; M: male; F: female; HBeAg: Hepatitis B e antigen; anti-HBe: antibodies against HBeAg; NP: not performed; ND: not detected.

## Data Availability

All relevant data are within the manuscript.
